# Exogenous Dopamine Application Promotes Alkali Tolerance of Apple Seedlings

**DOI:** 10.3390/plants8120580

**Published:** 2019-12-07

**Authors:** Xueyi Jiao, Yuxing Li, Xiuzhi Zhang, Chenlu Liu, Wei Liang, Chao Li, Fengwang Ma, Cuiying Li

**Affiliations:** State Key Laboratory of Crop Stress Biology for Arid Areas/Shaanxi Key Laboratory of Apple, College of Horticulture, Northwest A&F University, Yangling, Shanxi 712100, China; j1508102014@163.com (X.J.); lyx0707edu@163.com (Y.L.); zxz0124@yeah.net (X.Z.); lcl215719@163.com (C.L.); wei.liang@nwsuaf.edu.cn (W.L.); cl8609@nwsuaf.edu.cn (C.L.); fwm64@nwsuaf.edu.cn (F.M.)

**Keywords:** Malus, alkali stress, dopamine, antioxidant capacity

## Abstract

Arid and semiarid apple producing areas suffer from severe alkalinity of soil, which strongly affects the yield and quality of apples. Dopamine (DA) is involved in metabolic activities in response to abiotic stress in plants. To detect the effects of exogenous DA application on the adaption of apple (*Malus hupehensis*) seedlings to alkali stress and as a protection from oxidative stress, 0.1 mM DA was identified as the most suitable concentration by hydroponic culture. Further experimentation showed that the growth and photosynthesis of apple seedlings were significantly inhibited under alkali stress, and more reactive oxygen species accumulated, compared with control. However, exogenous DA application suppressed the loss of the plant height, root length, chlorophyll levels, and photosynthetic capacity of apple seedlings that were caused by alkali stress. In the leaves of alkali stressed seedlings, the catalase, superoxide dismutase, and peroxidase activities were lower and hydrogen peroxide and malondialdehyde levels were higher than in the untreated plants. The presence of DA significantly alleviated such effects of alkali stress. In addition, exogenous DA application increased the antioxidant capacity of apple seedlings under alkali stress by increasing the level of chlorogenic acid. These results are significant for improving the alkali tolerance of apple in apple-producing areas with alkalized soil.

## 1. Introduction

The loess plateau of Northwest China has a large diurnal temperature range and sufficient sunshine, both of which contribute to the production of high-quality apples; however, the climate is arid or semiarid. The low annual precipitation and the large evaporation in this area lead to the accumulation of alkali salts in the surface soil. Consequently, soil salinization is becoming increasingly severe, and the pH value of the soil exceeds 8.0 [1]. To promote the production of high-quality apples, agritechnological measures to improve the alkali tolerance of apple trees are of great importance. How plants respond to salt stress has been widely studied, but little attention has been focused on the response mechanisms of plants to alkali stress. Salt stress is mainly caused by neutral salt NaCl, whereas alkali stress is mainly caused by NaHCO_3_ and Na_2_CO_3_ [2,3]. The effects of alkali stress on plant growth mainly manifested in the damage of partial root function, decreases of water content and root activity, destruction of the photosynthetic system, and a decrease of photosynthetic pigment levels [3]. Previous studies have shown that alkali stress can significantly inhibit the growth of apple seedlings, damage plasma membrane, and cause a series of reductions of physiological functions [4,5,6].

When plants are subjected to environmental stresses, an effective reactive oxygen species (ROS) scavenging system is important. Excessive oxidation molecules, which accumulate in plants, can be removed in time, which maintains the normal oxidation state of cells. The scavenging mechanism of ROS can be divided into enzyme systems and nonenzymatic systems [7]. Superoxide anions (O_2_^−^) are catalyzed by superoxide dismutase (SOD) to produce hydrogen peroxide (H_2_O_2_). Peroxidase (POD) and catalase (CAT) can transform excess H_2_O_2_ into water [8]. In addition, phenols can also scavenge ROS and improve the antioxidant effect of plants [9]. Chlorogenic acid (CHA) forms the main component of polyphenols and is an antioxidant with the physiological activities of scavenging free radicals and antioxidation [10]. Exogenous CHA application can alleviate senescence, significantly reduce both the ethylene content and respiration rate, and increase firmness and the content of soluble solids [11].

Dopamine (DA) is a water-soluble antioxidant, and plays an important role in plant abiotic stress [12,13]. DA regulates a variety of metabolic activities in plant cells, such as active oxygen scavenging processes [14], plant sugar metabolism, regulation of ion permeability, and photophosphorylation of chloroplasts [15,16,17]. It can also interact with plant hormones and affect the growth and development of plants [18,19,20]. However, little is known how the effect of alkali stress guides the antioxidant capacity of apple seedlings.

Therefore, this study used *Malus hupehensis* (with apomixis characteristics) to study whether the effects of DA application could enhance the alkali stress tolerance of apple seedlings under hydroponic cultivation. In particular, the potential antioxidant properties of DA were investigated. The results of this study corroborate the value of exogenous DA application for the producing of apples in arid and semiarid areas.

## 2. Results

### 2.1. Screening of Suitable Dopamine Concentrations

**Figure 1:**
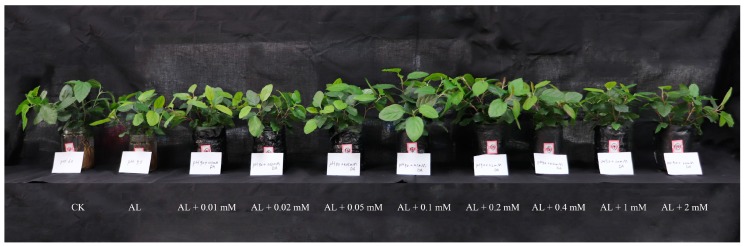
Phenotypes of apple seedlings treated by dopamine (DA) application under alkali stress for 15 days. CK: control (pH = 6.0); AL: alkali stress (pH = 9.0).

**Figure 2:**
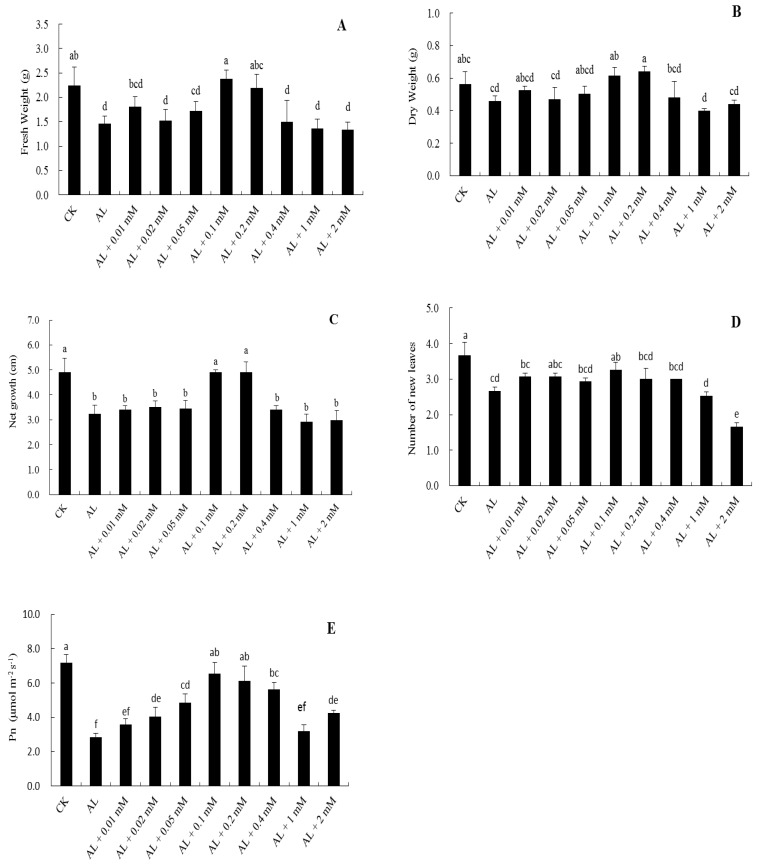
Effects of different concentrations of dopamine (DA) application on fresh weight (**A**), dry weight (**B**), net growth (**C**), number of new leaves (**D**), and net photosynthetic rate (Pn) (**E**) of apple seedlings under alkali stress for 15 days. Data are means ± SD (for Pn, *n* = 10; for the others, *n* = 20). Tukey’s multiple range test (two-way) based on analysis of variance (ANOVA) was chosen to analyze the data. Values with different letters are significantly different (*P* < 0.05). For information on treatments, please see Figure 1.

Seedlings grown to six to eight true leaves were precultured hydroponically in Hoagland nutrient solution for 15 days, and treated with an alkalinized (pH 9.0) nutrient solution for 15 days (AL), as in Materials and Methods. For control treatment (CK), seedlings were cultured in a neutral (pH 6.0) nutrient solution. DA was added to the nutrient solution on the first day of treatment. The seedlings treated with 0.1 mM DA were higher and their leaves were larger compared with those of other alkali stress treatments, and the new leaves were also greener, which were more similar to that of the control, as seen in Figure 1, Figure S1. Compared with control, alkali stress significantly decreased the fresh weight, dry weight, net growth, and number of new leaves, while the inhibitory effect was decreased after DA application. In particular, 0.1 mM DA and 0.2 mM DA could restore the growth of the seedlings under alkali stress to the control level, as seen in Figure 2A–D. The net photosynthesis rate (Pn) was significantly suppressed under alkali stress. In the presence of DA at 0.1–0.2 mM, the suppression of Pn was significantly smaller, as seen in Figure 2E. Therefore, 0.1 mM DA and 0.2 mM DA application had the most significant palliative effect.

### 2.2. Effects of Dopamine Application on Plant Growth

**Figure 3:**
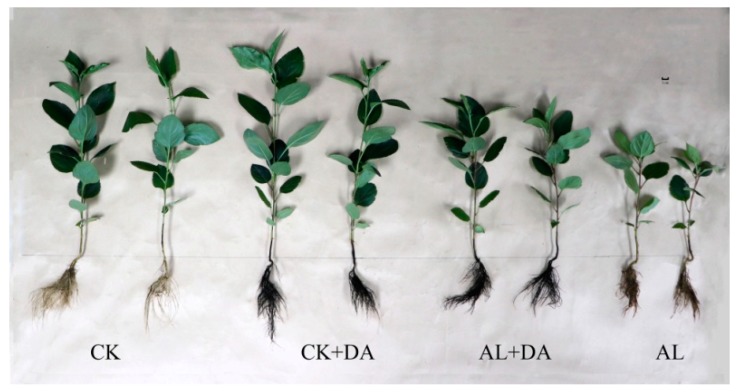
Phenotype of apple seedlings treated with dopamine (DA) application under alkali stress for 15 days. CK: control (pH = 6.0); CK+DA: control + 0.1 mM DA; AL+DA: alkali stress (pH = 9.0) + 0.1 mM DA; AL: alkali stress.

Compared with control, the growth of apple seedlings was significantly inhibited in response to alkali stress; however, after the application of exogenous DA, this inhibitory effect was significantly alleviated, and the leaves basically returned to a tender green color similar to that of the control, as seen in Figure 3, Figure S2.

**Figure 4:**
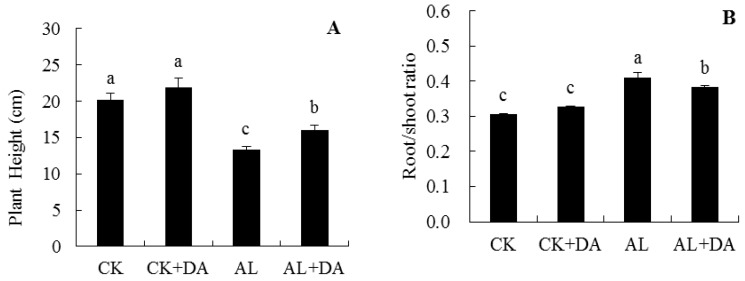
Effects of dopamine (DA) application on plant height (**A**) and root/shoot ratio (**B**) of apple seedlings under alkali stress for 15 days. Data are means ± SD (n = 15). Tukey’s multiple range test (two-way) based on analysis of variance (ANOVA) was chosen to analyze the data. Values with different letters are significantly different (*P*< 0.05). For information on treatments, please see Figure 3.

Under normal conditions, exogenous DA had no significant effect on plant height or root/shoot ratio of apple seedlings, as seen in Figure 4. The seedling height under alkali stress was 34.2% lower than the untreated control. Addition of DA significantly alleviated the growth suppression by alkali stress, as seen in Figure 4A. The root/shoot ratio of apple seedlings under alkali stress was 1.06 times higher than that of the control. Compared with alkali-stressed plants, the root/shoot ratio of apple seedlings decreased by 6.6% after addition of 0.1 mM DA, as seen in Figure 4B.

### 2.3. Effects of Dopamine Application on Root System Architecture

Root volume, forks, and root surface area were increased by DA treatment under control conditions, but exogenous DA had no significant effects on the root length, root diameter, and tips. After 15 days of alkali stress, compared with control, root length, tips, and forks significantly decreased by 32.8%, 44.9%, and 20.8%, respectively. There was no significant difference in root diam, root volume and root surface area between alkali stress and control. Exogenous DA application alleviated the inhibitory effects on the root length significantly. DA also showed a tendency to alleviate the inhibition on tips and forks although the difference between these parameters in the presence and the absence of DA were not significant, as seen in Table 1.

### 2.4. Effects of Dopamine Application on Chlorophyll Content and Fv/Fm

**Figure 5:**
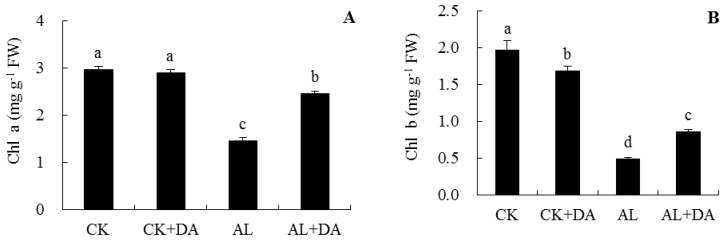

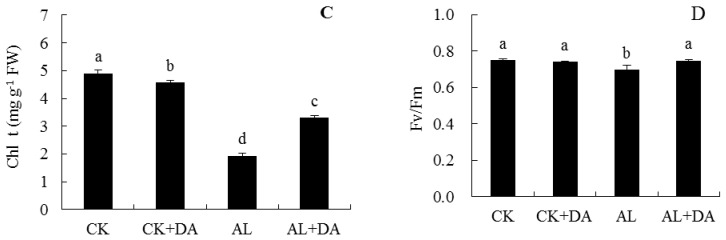
Effects of dopamine (DA) application on chlorophyll a (chl a, **A**), chlorophyll b (chl b, **B**), total chlorophyll (chl t, **C**), and Fv/Fm (**D**) of apple seedlings under alkali stress for 15 days. Data are means ± SD (for Fv/Fm, *n* = 15; for the others, *n* = 3). Tukey’s multiple range test (two-way) based on analysis of variance (ANOVA) was chosen to analyze the data. Values with different letters are significantly different (*P*< 0.05). For information on treatments, please see Figure 3.

Under normal conditions, exogenous DA had no significant difference in chlorophyll a (chl a) and Fv/F_m_, but exogenous DA significantly reduced chlorophyll b (chl b) and total chlorophyll (chl t) in leaves of apple seedlings, as seen in Figure 5. After 15 days of alkali stress, the contents of chl a, chl b, and chl t in leaves of apple seedlings were significantly lower than those of the control. They decreased by 50.8%, 75.1%, and 60.4%, respectively. Addition of DA suppressed the decreases in chl a, chl b, and accordingly chl t, as seen in Figure 5A–C. Under alkali stress, the Fv/F_m_ value in response to exogenous DA application was only 0.5% lower than that of the control, while the Fv/Fm value of alkali stress was 7.3% lower than that of the control, as seen in Figure 5D.

### 2.5. Effects of Dopamine Application on the Accumulation of Reactive Oxygen Species

**Figure 6:**
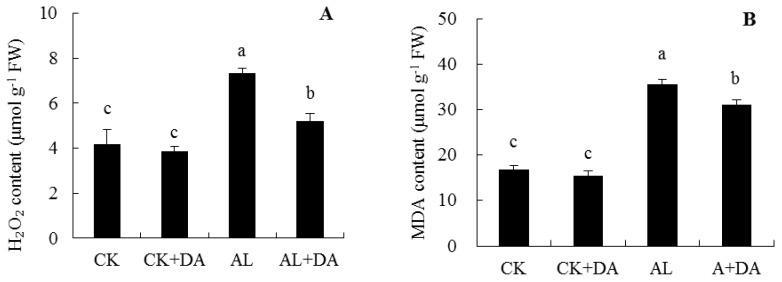

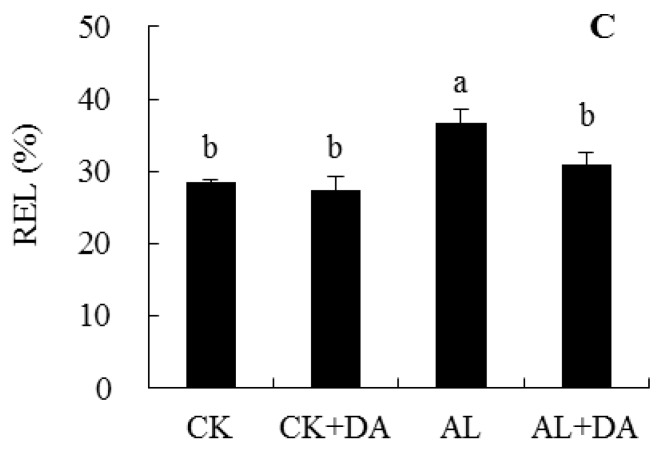
Effects of dopamine (DA) application on hydrogen peroxide (H_2_O_2_) (**A**), malondialdehyde (MDA) (**B**), and relative electrolyte leakage (REL) (**C**) in leaves of apple seedlings under alkali stress for 15 days. Data are means ± SD (*n* = 3). Tukey’s multiple range test (two-way) based on analysis of variance (ANOVA) was chosen to analyze the data. Values with different letters are significantly different (*P*< 0.05). For information on treatments, please see Figure 3.

Under normal conditions, exogenous DA caused no significant difference in H_2_O_2_ level or content of MDA and REL, as seen in Figure 6. Compared with control, alkali stress significantly increased the content of H_2_O_2_ in leaves of apple seedlings by 73.8%; however, exogenous DA application significantly suppressed the increase in the H_2_O_2_ level under alkali stress, which decreased by 28.9% compared with alkali stressed plants, which retained a relatively low H_2_O_2_ level, as seen in Figure 6A.

The content of MDA in leaves under alkali stress was 2.1 times higher than that of the control. Under alkali stress, DA application significantly suppressed the increase, as seen in Figure 6B.

Compared with control, after 15 days of alkali treatment, the REL in leaves of apple seedlings increased significantly (by 28.4%). Supplementation of DA to the seedlings efficiently prevented the alkali stress-induced increase in REL in leaves, as seen in Figure 6C. These results clearly indicate that exogenously added DA alleviated oxidative damage in seedlings under alkali stress.

### 2.6. Effects of Dopamine Application on Antioxidant Capacity

**Figure 7:**
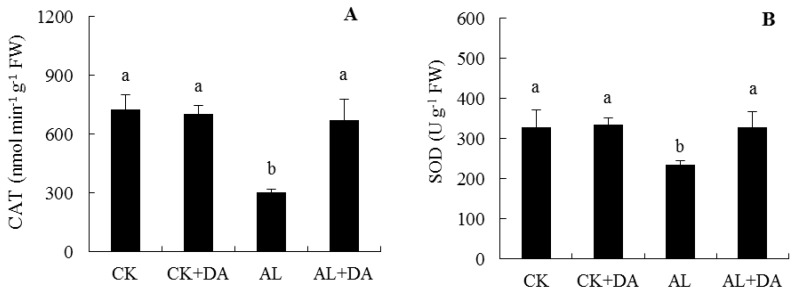

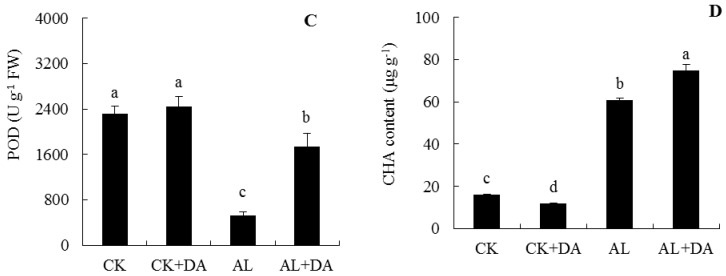
Effect of dopamine (DA) application on the activities of CAT (**A**), SOD (**B**), and POD (**C**) and chlorogenic acid (CHA) content (**D**) in leaves of apple seedlings under alkali stress for 15 days. Data are means ± SD (*n* = 3). Tukey’s multiple range test (two-way) based on analysis of variance (ANOVA) was chosen to analyze the data. Values with different letters are significantly different (*P*< 0.05). For information on treatments, please see Figure 3.

Under normal conditions, exogenous DA had no significant difference in activities of CAT, SOD, and POD, but exogenous DA significantly reduced the content of CHA in leaves of apple seedlings, as seen in Figure 7. The activities of CAT and SOD in leaves of apple seedlings under alkali stress were significantly lower than that of the control; however, for DA treatments, the activities of CAT and SOD were 2.2 and 1.4 times higher than those of alkali stressed plants, respectively, and the difference was significant, as seen in Figure 7A,B. Under alkali stress, DA application suppressed the decrease in the POD activity. The overall trend was consistent with that of both CAT and SOD, as seen in Figure 7C.

Exogenous DA application promoted the content of CHA to improve the antioxidant ability under alkali stress, as seen in Figure 7D. Compared with control, alkali stress increased the CHA content. At the same time, exogenous DA application significantly promoted the accumulation of CHA of apple seedlings, the content of which increased by 23.12% compared to alkali stress conditions.

### 2.7. Endogenous Dopamine Content

**Figure 8:**
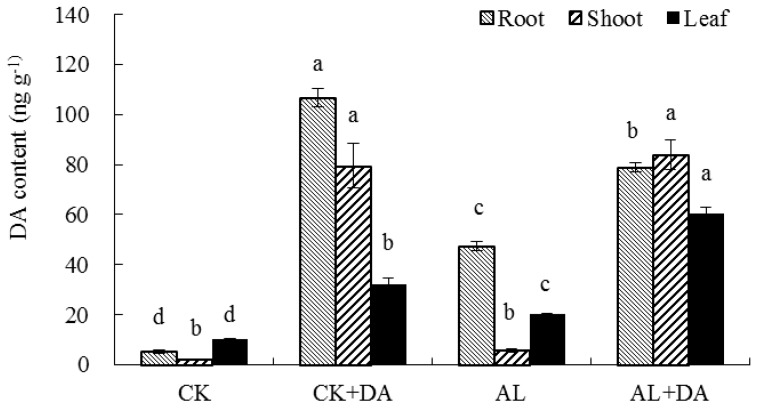
Effects of exogenous dopamine (DA) application on endogenous DA content of apple seedlings under alkali stress for 15 days. Data are means ± SD (*n* = 3). The different letters in the same style columns indicated significance at *P* < 0.05. Tukey’s multiple range test (two-way) based on analysis of variance (ANOVA) was chosen to analyze the data. Values with different letters are significantly different (*P* < 0.05). For information on treatments, please see Figure 3.

The DA contents in leaves, roots, and stems of apple seedlings treated with DA were 3.1, 20.3, and 37.3 times higher than those of the control, respectively. However, for seedlings under alkali stress, the DA contents in leaves, roots and stems were 1.9, 9.1, and 2.7 times higher than those of the control, respectively. It showed that alkali stress significantly promoted the accumulation of endogenous DA in leaves and roots of apple seedlings. Under alkali stress with 0.1 mM DA application, the DA contents in leaves, roots, and stems were 3.0, 1.7, and 14.4 times higher than those under alkali stress, respectively. Significant differences were found between the two treatments for each tissue, especially stems, as seen in Figure 8.

## 3. Discussion

This study was conducted to enhance our understanding of the physiological processes of apple seedlings in alkaline environments. DA was used to study its role during the alleviation of alkali stress. Although exogenous DA application can reduce the effects of drought stress, salt stress, and nutritional deficiency [12,13,21], the effect of DA on alkali stress has not been studied to date. Although alkali stress led to a decrease of many growth parameters, exogenous DA application significantly alleviated these inhibitory effects. Therefore, the physiological process of DA for improving the alkali tolerance in plants was detected.

### 3.1. Growth

Alkali stress exerts a significant inhibitory effect on the growth and development of plants [22]. In particular, it exerts a most severe effect on the root system [6]. The root architecture damage of *M. hupehensis* (which is an alkali-sensitive species) was stronger than that of *M. prunifolia* (which is alkali-tolerant) under alkali stress [23]. In this study, the root length, tips, and forks were decreased by alkali stress, while exogenous DA application could alleviate these inhibitions, as seen in Table 1. Furthermore, alkali stress inhibited the biomass, new leaf number, and plant height of apple seedlings, while exogenous application of 0.1 mM DA and 0.2 mM DA significantly alleviated this inhibition, as seen in Figure 2. This agrees with previous studies [4,24], which showed that exogenous DA application alleviated alkali stress.

### 3.2. Pn, Chlorophyll Content, and Fv/Fm

Among the many processes in which a stressful environment affects the plant physiological metabolism and thus suppresses plant growth, the effect on Pn was most severe, since it could directly change biomass [25]. Previous studies showed that the application of exogenous substances can increase the photosynthetic rate of plants under stress. The Pn of apple seedlings under salt stress was significantly increased by exogenous myo-inositol [26]. Exogenous γ-amino butyric acid (GABA) significantly suppressed the loss of the net photosynthetic rate of muskmelon seedlings caused by saline-alkali stress [27]. The results of the present study showed that Pn and the maximal photochemical efficiency of photosystem II (Fv/Fm) of leaves decreased significantly, while the concentration of intercellular CO_2_ increased significantly under alkali stress. However, 0.1 mM exogenous DA application could significantly alleviate the inhibitory effects, as seen in Figure 2E and Figure 5D.

The most obvious phenotype is the yellowing phenomenon of leaves in response to alkaline conditions, which showed a decrease of chlorophyll content [5,28]. In this study, compared with control, the contents of chl a, chl b, and chl t significantly decreased under alkali stress, as seen in Figure 5A–C. This may indicate damage of cell membrane permeability by alkali stress. Exogenous DA application alleviated these damages, as evidenced by their relatively lower REL values, as seen in Figure 6C.

### 3.3. Antioxidant System

ROS are intermediates that are produced by plants as part of their normal metabolic processes and do not normally damage cells. When plants are subjected to stress (heat stress, salt stress, or chilling stress), plants will accumulate large amounts of ROS and thus suffer oxidative damage [13,29,30]. At the same time, plant cells will scavenge ROS through their antioxidant system and reduce the cell membrane damage caused by stress. This study showed that SOD, CAT, and POD levels in apple leaves decreased significantly, while the H_2_O_2_ content increased under alkali stress. DA application restored these levels to almost the same level as those detected in the control, as seen in Figure 6A and Figure 7A–C). This might be attributed to the strong antioxidant capacity of DA, which is a water-soluble antioxidant. This phenomenon may also be because DA oxidation leads to the production of melanin, which is an effective scavenger of free radicals. This was similar to previous studies, where exogenous DA could increase the activities of SOD, CAT, and POD and suppressed the H_2_O_2_ increase in apple seedlings under salt stress [13].

Surprisingly, the CHA contents in apple leaves were found to significantly increase under alkali stress and exogenous DA application, as seen in Figure 7D. CHA is the main polyphenol representative of hydroxycinnamic acid, which is produced by the cinnamic acid pathway during aerobic inhalation [31]. CHA has antioxidant activity and can quench free radicals and ROS by providing electrons or hydrogen [32]. This might be because DA could fine-tune the stress response of organisms [24]. In our study, DA promoted the accumulation of CHA and improved the antioxidant capacity of apple seedlings under alkali stress.

### 3.4. Dopamine Content

Exogenous DA application can improve the stress resistance of plants. Previous studies showed that plant amine oxidase oxidizes monoamine to aldehyde and then participates in amine degradation [33]. One of the most important chemical reactions is that DA produces melanin through the oxidation of lipoxygenase. Under dark conditions, after injury, the content of catecholamine in purslane increased and the content of DA in cactus increased [34]. Drought or ultraviolet treatment increased DA levels in potato tubers [35]. The present study showed that alkali stress significantly increased the DA content in roots and leaves compared with the control, as seen in Figure 8, which was consistent with previous studies [36]. This might be the result of DA self-regulation to increase the adaptability of plants to stress. Although the contents of DA in the stems after alkali stress did not significantly differ from that of the control, this might be because alkali stress mainly manifested in the roots and leaves and exerted little damage to the stem. Therefore, exogenous DA application promoted the secretion of endogenous DA in apple seedlings under alkali stress, and the contents of DA in roots, stems, and leaves increased significantly, as seen in Figure 8. This indicated that DA could improve the tolerance of apple seedlings to alkali stress to alleviate the inhibitory effect this alkali stress imposes on apple seedlings.

## 4. Materials and Methods

### 4.1. Plant Materials

*Malus hupehensis* has apomixis characteristic; therefore, the seedlings showed good consistency of growth. In this experiment, the seeds of *M. hupehensis* were collected at Pingyi of Shandong province, China, and were treated by stratification in refrigerator at the fresh-keeping layer for about 40 days. Then, they were seeded into 50-hole plates (using peat substrate). When the seedlings grew to having three true leaves, Hoagland nutrient solution (pH = 6.0 ± 0.1, pH controlled with H_2_SO_4_) [37] was poured into the water twice a week, during which the seedlings were watered normally. When seedlings had grown to six to eight true leaves, the uniform seedlings were planted in plastic basins with 5.5 L Hoagland nutrient solution for 15 days of hydroponic preculture. The light-exposure time was 14 h within a 24 h period, and the light intensity was 8000–9000 Lx, the temperature was 22–24 °C/15–18 °C (day/night). During their culture, an air pump (ACO-318, Guangdong Haili Group Co., Ltd., Guangzhou, China) was used for regular ventilation (ventilated for 1 h, stopping for 30 min), and the solution was refreshed every five days.

### 4.2. Screening of Suitable Concentrations of Exogenous Dopamine Application

After 15 days of preculture, the seedlings were randomly divided into 10 groups with 20 plants per group. One group was control (CK), in which the pH of the nutrient solution was maintained at 6.0 ± 0.1; however, the others were under alkali conditions (pH = 9.0 ± 0.1, AL). Based on previous work, to simulate alkaline conditions, 1 M Na_2_CO_3_ and 1 M NaHCO_3_ (1:1, v/v) were used to adjust the pH of the nutrient solution to 9.0 ± 0.1. The groups under alkali stress were added the different DA concentrations of 0.01, 0.02, 0.05, 0.10, 0.20, 0.40, 1.00, and 2.00 mM into nutrient solution, respectively. The rest of the culture conditions remained identical, and the nutrient solution was renewed every five days. 

Prior to treatment, the youngest leaves of 20 seedlings were marked. Then, the number of new leaves was counted at the end of this experimental stage. The height of such marked plants was measured before treatment and after 15 days of alkali stress, which was used to calculate the net growth of the plant as indicated by the height after treatment minus the height before treatment. Ten seedlings of each treatment were randomly selected to assess both the fresh weight and dry weight of whole plants. Whole seedlings were fixed at 105 °C for 15 min, then dried at 65 °C for 48 h until they remained at a constant weight, which was defined as the dry weight. After 15 days of treatment, 10 seedlings were randomly chosen to measure the net photosynthetic rate (Pn) by portable photosynthesis meter Li6400 (LICOR, Huntington Beach, CA, USA) [21]. The Pn was determined between 9:00 and 11:00 a.m. All measurements were performed at 1000 μmol photons m^−2^ s^−1^ and a constant airflow rate of 500 μmol s^−1^. The cuvette CO_2_ concentration was set at 400 μmol CO_2_ mol^−1^ air. A suitable concentration of exogenous DA was selected using these indicators.

### 4.3. Mitigation Effect of Exogenous Dopamine Application

Based on the results of concentration screening, further research was conducted using 0.1 mM DA. Six hundred uniformed seedlings were randomly divided into 12 basins, with 50 seedlings per basins for 15 days of preculture. The nutrient solution was renewed every five days. After 15 days, the seedlings were randomly divided into four groups. The pH of the nutrient solution of two groups was maintained at 6.0 ± 0.1 (CK), to one of which was added 0.1 mM DA. The other two groups were under alkali conditions (AL), the pH of which was adjusted to 9.0 ± 0.1 by 1 M Na_2_CO_3_ and 1 M NaHCO_3_ (1:1, v/v), and to one of which was added 0.1 mM DA. The treatment lasted for 15 days. During this treatment, the pH value of the solution was measured by a pH meter (ST2100, Ohaus Instrument (Changzhou) co., Ltd., Changzhou, China) and was adjusted every day. All other culture conditions were identical to those of the preculture.

To further assess the promoting effects of exogenous DA application on the alkali tolerance and the protection of the antioxidant capacity, indicators related to plant growth, ROS damage, antioxidases, and antioxidants were measured after 15 days of treatment. The plant height and dry weight of both roots and shoots of 15 seedlings were determined, and the root/shoot ratios were calculated. Chlorophyll was extracted by 80% acetone, and the content was determined using Arnon’s method [38]. Chlorophyll fluorescence (Fv/Fm) was measured with a FC 800-O (Photon Systems Instruments, Brno, Czech Republic). The leaves to be tested were dark adapted for 1 h and measurements were conducted at 10:00 p.m. following Liang et al. [21]. Minimum fluorescence (F_0_) was measured under weak modulated irradiation (<0.1 µmol m^−2^ s^−1^). A 600 ms saturating flash (>7000 µmol m^−2^ s^−1^) was applied to measure the maximum chlorophyll fluorescence yield (Fm). Fv/Fm was calculated as (Fm − F_0_) / Fm. Relative electrolyte leakage (REL) was measured by the Dionisio-Sese and Tobita’s method [39], and the malondialdehyde (MDA) content was measured by the TBA method [40]. 

At the end of treatment, the roots of 10 seedlings were randomly selected from each group and scanned with a root scanner (Epson Perfection V700 Photo, Seiko Epson Corp, Suwa, Japan), the results of which were analyzed with the WinRHIZO root analysis system (Regent instruments, Quebec, Canada).

The content of H_2_O_2_ and the activities of CAT, SOD, and POD in leaves were determined separately by kits (COMIN, Keming Biotechnology Co. Ltd., Suzhou, China). CAT activity was expressed as the reduction of H_2_O_2_ per min per g of fresh weight (nmol min^−1^ g^−1^ FW); however, SOD activity and POD activity were expressed as units per gram fresh weigh (U g^−1^ FW). One unit (U) of SOD activity was defined as the activity of SOD when the inhibition of the xanthine oxidase coupling reaction system was 50%, while one unit (U) of POD activity was defined as the variation of 0.01 in A470 per mL reaction system.

The content of endogenous DA was determined by Kim et al. [41]. Leaf, shoot, and root tissues (0.10 g) were ground and extracted with 1.5 mL 90% methanol, then ultrasonically crushed for 10 min. The extract was centrifuged at 4 °C and 12,000 rpm for 10 min. The supernatant was filtered by a 0.22 µm water filter membrane. The filtered liquid was tested by liquid chromatography–mass spectrometry (QTRAP5500, AB Sciex Pret. Ltd., Framingham, MA, USA), with ammonium formate, acetonitrile, and water as the mobile phase.

The content of CHA was extracted according to Zhang et al. [42]. Fresh leaves (0.1 g) were homogenized with 1 mL (50:48:2 = methanol:ddH_2_O:formic acid), and the mixture was oscillatory vortex extracted for 5 min, and then centrifuged for 20 min at 4 °C and 10,000 rpm. The supernatant was absorbed and filtered by a 0.22 µm water syringe filter for measurement. The filtered liquid was tested by ATRAP 5500. Mobile phase A was 0.1% formic acid water, and mobile phase B was pure methanol. The flow rate was 0.3 mL min^−1^, and 5 µL was used as sample injection volume.

### 4.4. Statistical Analysis

All data were statistically analyzed with Microsoft Excel 2010 and SPSS 21.0 statistical analysis software (*p* < 0. 05). Tukey’s multiple range test (two-way) based on analysis of variance (ANOVA) was chosen to analyze the data.

## 5. Conclusions

The results showed that exogenous DA application (0.1 mM) effectively alleviated the alkali stress that was manifested as increased biomass accretion, net photosynthetic rate, and chlorophyll content, as well as in changes of root morphology. Such improvement of growth and development under stress conditions may have resulted from the enhanced antioxidant capacity of apple seedlings treated with DA, in which the increased activity of antioxidant enzymes and intensified accumulation of CHA accompanied by decrease of ROS production were determined. Our findings showed the positive role of DA in alkali resistance and provide new opportunities for DA application in agricultural production.

## Figures and Tables

**Table 1:** Effects of dopamine (DA) application on the root architecture of apple seedlings under alkali stress.

	CK	CK+DA	AL	AL+DA
Root Length (cm)	540.24 ± 77.15ab	580.60 ± 33.75a	363.19 ± 14.03d	456.95 ± 23.93bc
Root Diameter (mm)	0.57 ± 0.02b	0.60 ± 0.05ab	0.59 ± 0.04ab	0.70 ± 0.05a
Root Volume (cm^3^)	1.38 ± 0.07b	2.38 ± 0.42a	1.33 ± 0.14b	2.42 ± 0.40a
Tips	1031.75 ± 25.82a	1011.00 ± 100.33a	569.00 ± 50.01b	817.25 ± 191.62ab
Forks	2418.00 ± 244.71b	3272.33 ± 211.76a	1914.00 ± 99.27c	2406.67 ± 38.39bc
Root Surface Area (cm^2^)	65.64 ± 5.48bc	89.91 ± 4.94a	61.76 ± 3.11c	76.85 ± 5.99b

Data are means ± SD (*n* = 10). Tukey’s multiple range test (two-way) based on analysis of variance (ANOVA) was chosen to analyze the data. Values with different letters of each index are signiﬁcantly different (*P* < 0.05). For information on treatments, please see Figure 3.

## Supplementary Materials

The following are available online at https://www.mdpi.com/2223-7747/8/12/580/s1, Figure S1: Phenotype of leaves of apple seedlings treated with dopamine (DA) of different concentration under alkali stress for 15 days, Figure S2: Phenotypes of leaves of apple seedlings treated with dopamine (DA) under alkali stress for 15 days. For information on treatments.
