# Supplementary material for: Exogenous Dopamine Application Promotes Alkali Tolerance of Apple Seedlings
Source: Plants (Basel). 2019 Dec 7;8(12):580. doi: 10.3390/plants8120580 (PMC6963653; doi:10.3390/plants8120580)
Supplement: Supplementary file 1 [file plants-08-00580-s001.pdf]

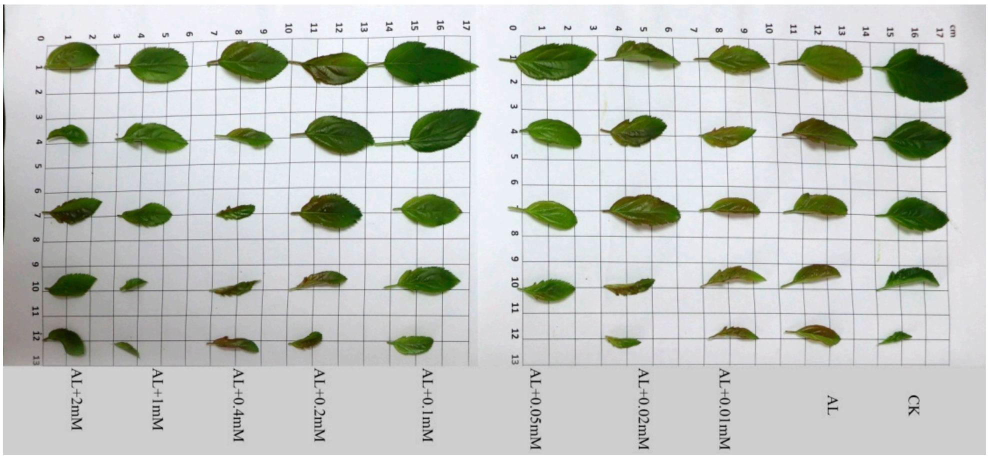

Figure S1. Phenotype of leaves of apple seedlings treated with dopamine (DA) of different concentration under alkali stress for 15 days. For information on treatments, please see Figure 1.

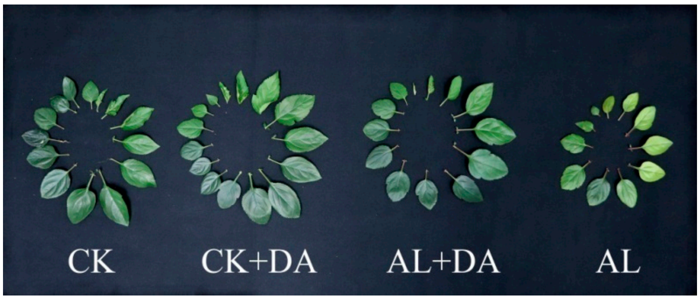

Figure S2. Phenotypes of leaves of apple seedlings treated with dopamine (DA) under alkali stress for 15 days. For information on treatments, please see Figure 3.
